# Impact of a change from A–F grading to honors/pass/fail grading on academic performance at Yonsei University College of Medicine in Korea: a cross-sectional serial mediation analysis

**DOI:** 10.3352/jeehp.2024.21.20

**Published:** 2024-08-16

**Authors:** Min-Kyeong Kim, Hae Won Kim

**Affiliations:** 1 Institute of Behavioral Science in Medicine, Yonsei University College of Medicine, Seoul, Korea; 2 Department of Medical Education, Yonsei University College of Medicine, Seoul, Korea; Hallym University, Korea

**Keywords:** Academic performance, Medical students, Mediation analysis, Motivation, Republic of Korea

## Abstract

**Purpose:**

This study aimed to explore how the grading system affected medical students’ academic performance based on their perceptions of the learning environment and intrinsic motivation in the context of changing from norm-referenced A–F grading to criterion-referenced honors/pass/fail grading.

**Methods:**

The study involved 238 second-year medical students from 2014 (n=127, A–F grading) and 2015 (n=111, honors/pass/fail grading) at Yonsei University College of Medicine in Korea. Scores on the Dundee Ready Education Environment Measure, the Academic Motivation Scale, and the Basic Medical Science Examination were used to measure overall learning environment perceptions, intrinsic motivation, and academic performance, respectively. Serial mediation analysis was conducted to examine the pathways between the grading system and academic performance, focusing on the mediating roles of student perceptions and intrinsic motivation.

**Results:**

The honors/pass/fail grading class students reported more positive perceptions of the learning environment, higher intrinsic motivation, and better academic performance than the A–F grading class students. Mediation analysis demonstrated a serial mediation effect between the grading system and academic performance through learning environment perceptions and intrinsic motivation. Student perceptions and intrinsic motivation did not independently mediate the relationship between the grading system and performance.

**Conclusion:**

Reducing the number of grades and eliminating rank-based grading might have created an affirming learning environment that fulfills basic psychological needs and reinforces the intrinsic motivation linked to academic performance. The cumulative effect of these 2 mediators suggests that a comprehensive approach should be used to understand student performance.

## Graphical abstract

[Fig f2-jeehp-21-20]

## Introduction

### Background/rationale

Pass/fail grading in medical schools has received considerable attention because of its beneficial effect on students’ emotional well-being, without compromising their academic performance [1]. A contributor to these favorable academic outcomes might be students’ perceptions of their learning environment, which have been suggested to affect academic performance [2]. Since the pass/fail grading system helps students perceive the learning environment as supportive [3], these perceptions might contribute to their performance. Another potential factor associated with better performance is intrinsic motivation, which emerges from personal enjoyment or curiosity [4]. Unlike the detrimental effects of hierarchical grades, pass/fail grading has been suggested to foster intrinsic motivation [5]. Furthermore, students’ perceptions of their learning environment are correlated to intrinsic motivation [6] and are an essential antecedent for students’ motivation [7]. These results implied a cascading link between the grading system and academic performance in medical students, mediated by 2 factors, but further research is needed to explore this mechanism.

The known benefits of pass/fail grading might not simply be due to reducing the number of grades, but also to the characteristics of criterion-referenced assessments. Pass/fail grading implies a shift to a criterion-referenced standard setting through which students are compared against a clear standard and not against the performance of others. Therefore, the use of 3 or more categories based on a criterion, instead of only 2 categories (pass/fail), might yield positive results compared with relative rank-based tiers. Considering the value of criterion-referenced assessment, Yonsei University College of Medicine (YUCM) in Korea introduced a new grading system in 2014, changing from norm-referenced A–F grading to criterion-referenced honors/pass/fail grading. The new system, with fewer grades and criterion-referenced standards, might influence students’ learning in similar ways to what has occurred with pass/fail grading. However, there is a scarcity of relevant studies on this topic.

### Objectives

This study aimed to investigate how grading systems affect academic performance based on students’ perceptions of their learning environment and motivation. Drawing upon the findings of previous studies, we propose a serial mediation model between honors/pass/fail grading and academic performance. We hypothesized that: (1) students’ perceptions of the learning environment would mediate the relationship between the grading system and academic performance; (2) intrinsic motivation would mediate the relationship between the grading system and academic performance; and (3) learning environment perceptions and intrinsic motivation would serially mediate the relationship between the grading system and academic performance.

## Methods

### Ethics statement

The Institutional Review Board (IRB) of Severance Hospital, Yonsei University Health Systems, approved this study using data obtained from normal educational practice in commonly accepted educational settings; thus, it was considered as an IRB-exempt study (IRB no., Y-2020-0201). Therefore, the requirement for informed consent was waived.

### Study design

We adopted a cross-sectional study design to compare students’ survey responses and examination scores between before and after the grading system was changed.

### Setting

In 2014, YUCM in Korea introduced a new grading system, moving from a norm-referenced assessment using A–F letter grading (A+, A, A-, B+, B, B-, C+, C, C-, D+, D, D-, F) to a criterion-referenced assessment using honors/pass/fail grading. Under the new system, the core mandatory courses that all students take use a 3-tiered grading system, and elective courses use a 2-tiered (pass/fail) grading system. This change was the first attempt among 40 medical schools in Korea and was an innovative endeavor in the highly competitive Korean medical education context. As there were concerns about the possibility of compromised academic performance due to the removal of ranks before introducing the criterion-referenced honors/pass/fail grading system, the relationship between the new grading system and academic performance was perceived as a critical issue.

### Participants

This study included 238 second-year medical students from 2014 (n=127; A–F grading) and 2015 (n=111; honors/pass/fail grading). The former was the last cohort graded under the norm-referenced A–F grading system, and the latter was the first cohort to experience the new criterion-referenced 3-tiered grading system. The Department of Medical Education at YUCM conducted routine surveys on students at the end of each year to assess and ensure educational quality. Furthermore, upon completing the second academic year, corresponding to the end of the preclinical curriculum, students took the Basic Medical Science Examination as part of their requirements. Data from both the surveys and the examinations were used.

### Variables

The demographic characteristics of the study participants, including age, gender, and medical school entrance type (undergraduate entry: 6-year program; graduate entry: 4-year program), were analyzed in this study. For learning-related characteristics, we measured learning environment perceptions, intrinsic motivation, and academic performance in medical students.

### Data sources/measurements

#### Learning environment perceptions

Students’ perceptions of the learning environment were assessed using the Dundee Ready Education Environment Measure (DREEM) [8]. The DREEM comprises 50 items scored from 0 (strongly disagree) to 4 (strongly agree). The 9 negative statements are scored inversely. The DREEM measures students’ perceptions of learning, teachers, atmosphere, academic self, and social self. This scale can be analyzed in terms of the scores for individual items, 5 subscales, and all items, and a higher score signifies a more positive perception of the assessed construct. In this study, we used the total DREEM score as an indicator of overall learning environment perceptions. Cronbach’s α for the total scale was 0.864.

#### Intrinsic motivation

The Academic Motivation Scale (AMS) was used to assess intrinsic motivation for learning [9]. The AMS comprises 7 subscales that measure 3 types of intrinsic motivation (to know, accomplish things, and experience stimulation), 3 forms of extrinsic motivation (identified, introjected, and external regulation), and amotivation. Each AMS subscale comprises 4 items rated on a 7-point scale ranging from 1 (does not correspond at all) to 7 (corresponds exactly). In this study, we used the sum of the 3 intrinsic motivation subscale scores, with higher scores indicating a greater desire to engage in an activity based on intrinsic motivation. Cronbach’s α for this domain was 0.946.

#### Academic performance

The Basic Medical Science Examination scores, administered by the Korea Association of Medical Colleges, were used to measure students’ academic performance. This examination is a comprehensive and objective test of the medical knowledge of anatomy, biochemistry, physiology, pharmacology, microbiology, parasitology, and pathology. The examination included 260 multiple-choice items, and a score of 1 point was assigned for each correct answer. In this study, the percentage of correct scores was used for the analysis.

Raw and coding data are available at Dataset 1.

### Bias

No selection bias was observed in this study.

### Study size

The sample size was not estimated *a priori* because this study was conducted retrospectively using data collected from normal educational practices.

### Statistical methods

**Fig. 1.**
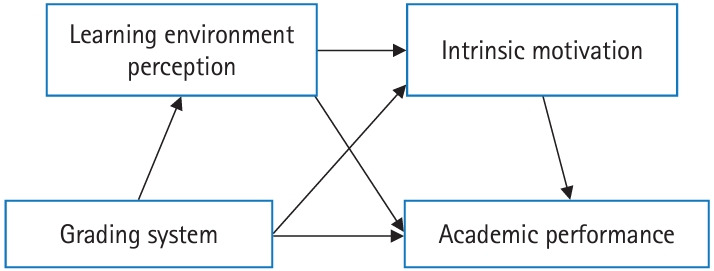
Conceptual model of the serial mediation effect of learning environment perceptions and intrinsic motivation on the relationship between the grading system and academic performance.

All statistical analyses were performed using R software ver. 4.0.2 (https://www.r-project.org). A 2-way imputation method was implemented to impute the missing item scores for learning environment perceptions and intrinsic motivation [10]. Each of the 2 scales, DREEM and AMS, had 0.9% of missing values. The R package “miceadds” was used for 2-way imputation (Supplement 1). To compare the characteristics of the A–F grading and honors/pass/fail grading classes, we conducted the independent sample t-test and chi-square test for continuous and categorical variables, respectively. Pearson correlation analysis was performed to explore the relationship between continuous variables, while the point biserial correlation method was used to measure the associations between continuous and binary variables. Next, the serial mediation effect of perceptions of the learning environment and intrinsic motivation on the relationship between the grading system and academic performance was examined. Fig. 1 presents the conceptual model. The PROCESS macro (model 6) was used to examine the indirect effect based on a 95% bootstrapped confidence interval (CI) with 10,000 resamplings. If the interval did not contain 0, a significant indirect effect was considered to have been shown. Participants’ gender and type of medical school entrance were controlled in the model.

## Results

### Participants

Table 1 shows the demographic and learning-related characteristics of the participants. The mean age of the 2 groups did not significantly differ, and there were no statistical differences in gender or entrance type distribution. Regarding learning-related characteristics, the honors/pass/fail grading class perceived the learning environment more positively and scored significantly higher on intrinsic motivation. The academic performance of the groups was different, with the honors/pass/fail grading class demonstrating higher performance on the Basic Medical Science Examination.

### Main results

Table 2 presents the correlation matrices for the study variables. All variables were positively correlated. Table 3 shows the coefficients in the hypothetical model. The honors/pass/fail grading class predicted better perceptions of the learning environment (unstandardized coefficient [β]=10.685, P<0.001). In addition, the grading system and perceptions of the learning environment were significant predictors of intrinsic motivation (β=3.453, P=0.033; β=0.242, P<0.001). Finally, the grading system and intrinsic motivation predicted academic performance (β=2.417, P=0.046; β=0.114, P=0.019), whereas learning environment perceptions did not. Thus, the honors/pass/fail grading class predicted more positive perceptions of the learning environment, which in turn predicted higher intrinsic motivation and better academic performance.

Table 4 presents the indirect effects estimated using bootstrapping. Learning environment perceptions and intrinsic motivation sequentially mediated the association between the grading system and academic performance (effect, 0.295; 95% bootstrapped CI, 0.021 to 0.665). However, there were no significant indirect effects when only learning environment perceptions or intrinsic motivation was used as a mediator (effect, 0.136; 95% bootstrapped CI, -0.492 to 0.733; effect, 0.395; 95% bootstrapped CI, -0.012 to 1.046, respectively). In addition, the effect of the grading system on performance was independent of learning environment perceptions or intrinsic motivation (β=2.417, P=0.046).

## Discussion

### Key results

This study sought to identify the mechanisms that might impact the academic performance of medical students during the transition from a norm-referenced A–F grading system to criterion-referenced honors/pass/fail grading. Our results showed a direct effect of the honors/pass/fail grading system on academic performance and a serial mediation effect in which the grading system indirectly influenced performance through perceptions of the learning environment and intrinsic motivation. Interestingly, the indirect effect of simple mediation via either mediator between the assessment system and student performance was not statistically significant.

### Interpretation

In this case, a change in the grading system improved students’ perceptions of the learning environment, subsequently increasing intrinsic motivation and, in turn, enhancing academic performance. This close relationship between intrinsic motivation and learning environment perceptions, which has been noted in the literature and supported in this study, might be explained by self-determination theory, an established framework for understanding motivation [11]. This theory emphasizes that fulfilling basic psychological needs (autonomy, competence, and relatedness) is fundamental to building intrinsic motivation. When viewed through the lens of self-determination theory, changing the grading system from the norm-referenced 13-category system to criterion-referenced honors/pass/fail grading might have had certain advantages [12]: (1) the removal of relative rank-based grades might have allowed students to pursue learning for curiosity and interest, and thus have increased their autonomy relating to their studies; (2) the implementation of a structured system in which the students could be assessed as having achieved enough when they met specific criteria could have inspired a sense of competence; and (3) increased relatedness might have been facilitated by alleviating competition and fostering a collaborative climate.

Collectively, our results suggest that the grading system transition provided a favorable learning environment for the students, as captured by their more positive perceptions, and resulted in higher intrinsic motivation, which led to better academic performance.

### Comparison with previous studies

Previous studies found benefits in learning environment perceptions [13] and intrinsic motivation [5] within pass/fail grading, and this study of the honors/pass/fail grading system reported these benefits as well. Furthermore, our study showed significant correlations between perceptions, intrinsic motivation, and academic performance in line with previous research [2,4,6,7]. However, perceptions of the learning environment were not identified as a valid variable for academic performance in the multivariable regression model of this study. These results imply that the independent effect of learning environment perceptions was diminished due to interactions with other variables. The results of the serial mediation analysis, which showed that learning environment perceptions and intrinsic motivation were not the sole mediators but sequential mediators, further confirmed their combined role in medical students’ performance.

### Limitations/generalizability

These findings broaden our understanding of how a change in the grading system could affect the academic performance of medical students. Despite its strengths, this study had several limitations. First, it examined only one cohort of students for each grading system at a single center. Therefore, the results might not be generalizable to other cohorts, settings, or populations. Second, the use of self-report questionnaires was a limitation in ensuring the findings’ validity. A mixed approach involving quantitative and qualitative measures would be helpful in triangulating our findings and further clarifying the underlying processes and mechanisms. Third, we could not exclude the influence of factors other than students’ perceptions of the learning environment and motivation in mediating the relationship between the grading system and academic performance. Fourth, an issue not addressed in this study was whether honors/pass/fail grading differentially affected students’ academic performance and mental health. Inconsistent findings have been reported on this matter, with some studies supporting the elimination of honors grades for the sake of well-being [14], while the distinction/pass/fail grading system has been reported to be beneficial relating to perceived stress [1]. However, note that such inconsistencies could have stemmed from the use of different control groups, since the former study focused on the pass/fail grading system as the comparison group, whereas the latter study used traditional letter grades as control groups. Therefore, further studies are needed to understand the varying effects of the honors/pass/fail grading system on medical students’ learning and mental health. Moreover, research on student motivation and performance during clerkship and residency training is needed to illustrate the long-term effects of changes in the grading system.

### Suggestions

A direct link between the grading system and students’ performance in our model might suggest an independent influence of the grading system or the presence of other mediating factors. Further studies are required to understand the comprehensive effects of honors/pass/fail grading and identify additional paths through which the grading system and medical students’ academic performance relate to each other. Although this study did not address extrinsic motivation, it would be necessary to understand its role in honors/pass/fail grading. An extensive meta-analysis found that intrinsic motivation and extrinsic incentives jointly affected performance and did not work in opposition to each other [15]. It was possible that an honors grade served as an external reward, and favorable academic performance in our study could have been affected by both intrinsic and extrinsic motivation. Thus, there might be various paths linking honors/pass/fail grading to academic performance that need to be further explained.

### Conclusion

Our results indicated that a change from a norm-referenced A–F grading to an honors/pass/fail grading system was associated with more positive perceptions of the learning environment and higher intrinsic motivation in the early years of medical education. The removal of rank-based evaluations might have fostered a positive learning environment that enhanced students’ intrinsic motivation for learning, which could be related to the fact that academic performance did not decline.

## Figures and Tables

**Figure f2-jeehp-21-20:**
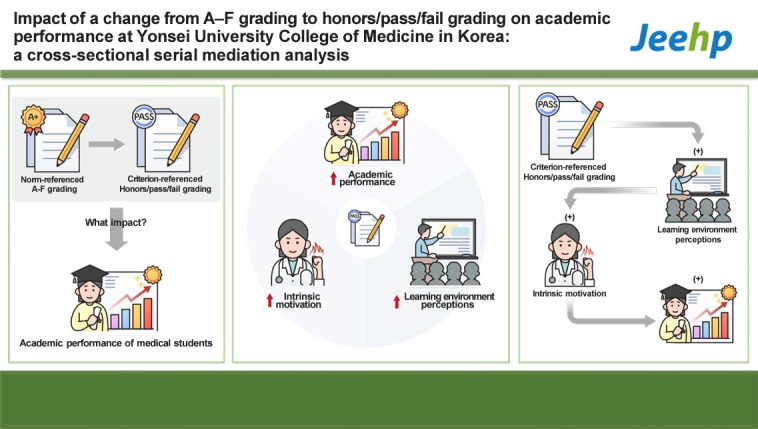


**Table 1.** Demographic and learning-related characteristics of participants

Characteristic	A–F grading class (n=127)	Honors/pass/fail grading class (n=111)	t or χ^2^	P-value
Age (yr)	23.72±2.21	23.39±2.09	1.203	0.230
Gender			0.195	0.659
Male	88 (69.3)	73 (65.8)		
Female	39 (30.7)	38 (34.2)		
Type of medical school entrance			0.003	0.959
Undergraduate-entry	69 (54.3)	59 (53.2)		
Graduate-entry	58 (45.7)	52 (46.8)		
Learning environment perceptions (range, 0–200)	110.81±22.46	121.72±24.68	-3.569	<0.001
Intrinsic motivation (range, 12–84)	48.93±13.55	55.11±13.43	-3.524	<0.001
Basic Medical Science Examination scores (range, 0–100)	48.76±9.51	52.00±8.54	-2.749	0.006

Values are presented as mean±standard deviation or number (%).

**Table 2.** Correlations between study variables

Variables by no.	1	2	3	4
1. Grading system (reference: A–F grading class)	1			
2. Learning environment perceptions	0.226^a)^	1		
3. Intrinsic motivation	0.224^a)^	0.468^a)^	1	
4. Academic performance	0.176^b)^	0.146^b)^	0.224^a)^	1

a) Correlation was significant at the 0.001 level.

b) Correlation was significant at the 0.05 level.

**Table 3.** Regression analysis of study variables in the serial mediation model

Explanatory variables	Outcome variables								
	Learning environment perception			Intrinsic motivation			Academic performance		
	β	SE	P-value	β	SE	P-value	β	SE	P-value
Gender	5.125	3.373	0.130	1.571	1.751	0.371	-1.266	1.296	0.330
Entrance type	3.702	3.163	0.243	2.825	1.638	0.086	1.812	1.219	0.138
Grading system (reference: A–F grading class)	10.685	3.039	<0.001	3.453	1.610	0.033	2.417	1.202	0.046
Learning environment perceptions				0.242	0.034	<0.001	0.013	0.028	0.644
Intrinsic motivation							0.114	0.048	0.019
R^2^	0.071			0.249			0.078		
F	5.987			19.346			3.920		
P-value	<0.001			<0.001			0.020		

SE, standard error.

**Table 4.** Mediating effects of learning environment perceptions and intrinsic motivation on the relationship between grading system and academic performance

Pathway	Effect	Boot SE	95% bootstrapped CI
Grading system → learning environment perceptions → academic performance	0.136	0.306	-0.492 to 0.733
Grading system → intrinsic motivation → academic performance	0.395	0.277	-0.012 to 1.046
Grading system → learning environment perceptions → intrinsic motivation → academic performance	0.295	0.167	0.021 to 0.665

SE, standard error; CI, confidence interval.

## Footnotes

**Data availability**
Data files are available from Harvard Dataverse: https://doi.org/10.7910/DVN/ZINJH6 Dataset 1. Raw response data from 238 participants and imputed coding data used to identify the serial mediation effect between the grading system and medical students’ performance in Korea.

## Supplementary materials

Supplementary files are available from Harvard Dataverse: https://doi.org/10.7910/DVN/ZINJH6

Supplement 1. R code for the analyses, including serial mediation analysis.

Supplement 2. Audio recording of the abstract.
