# Erratum: Impact of a change from A–F grading to honors/pass/fail grading on academic performance at Yonsei University College of Medicine in Korea: a cross-sectional serial mediation analysis

**DOI:** 10.3352/jeehp.2024.21.35

**Published:** 2024-11-26

**Authors:** Editorial office, Journal of Educational Evaluation for Health Professions

**Affiliations:** Hallym University, Korea

After the publication of the original article [1], an error was identified in Fig. 1. Specifically, during the production process, the arrow in Fig. 1 was incorrectly reversed. The arrow, which should indicate the transition from “grading system” to “learning environment perception,” was incorrectly shown as moving from “learning environment perception” to “grading system.”

This issue has been corrected, and the updated figure has been replaced in the original article. The corrected figure is provided below for reference.[Fig f1-jeehp-21-35]

The editorial office apologizes for this oversight and appreciates the authors’ diligence in identifying the error.

## Figures and Tables

**Fig. 1. f1-jeehp-21-35:**
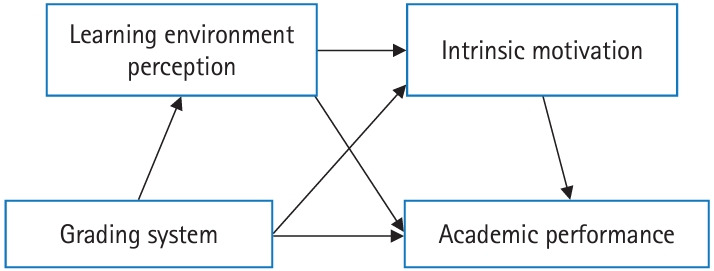
Conceptual model of the serial mediation effect of learning environment perceptions and intrinsic motivation on the relationship between the grading system and academic performance.
